# A novel homozygous *MPV17* mutation in two families with axonal sensorimotor polyneuropathy

**DOI:** 10.1186/s12883-015-0430-1

**Published:** 2015-10-05

**Authors:** Yu-Ri Choi, Young Bin Hong, Sung-Chul Jung, Ja Hyun Lee, Ye Jin Kim, Hyung Jun Park, Jinho Lee, Heasoo Koo, Ji-Su Lee, Dong Hwan Jwa, Namhee Jung, So-Youn Woo, Sang-Beom Kim, Ki Wha Chung, Byung-Ok Choi

**Affiliations:** Department of Biochemistry, Ewha Womans University School of Medicine, Seoul, Korea; Stem Cell & Regenerative Medicine Center, Samsung Medical Center, Seoul, Korea; Department of Biological Science, Kongju National University, 56 Gonjudaehak-ro, Gongju, Chungnam 314-701 Korea; Department of Neurology, Ewha Womans University School of Medicine, Seoul, Korea; Department of Neurology, Samsung Medical Center, Sungkyunkwan University School of Medicine, 81 Irwon-ro, Gangnam-Gu, Seoul, 135-710 Korea; Department of Pathology, Ewha Womans University School of Medicine, Seoul, Korea; Department of Microbiology, Ewha Womans University School of Medicine, Seoul, Korea; Department of Neurology, Kyung Hee University, College of Medicine, Seoul, Korea; Neuroscience center, Samsung Medical Center, Seoul, Korea

**Keywords:** Mitochondrial DNA depletion syndrome 6 (MTDPS6), *MPV17*, Navajo neurohepatopathy (NNH), Sensorimotor polyneuropathies, Whole exome sequencing (WES)

## Abstract

**Background:**

Mutations in *MPV17* cause the autosomal recessive disorder mitochondrial DNA depletion syndrome 6 (MTDPS6), also called Navajo neurohepatopathy (NNH). Clinical features of MTDPS6 is infantile onset of progressive liver failure with seldom development of progressive neurologic involvement.

**Methods:**

Whole exome sequencing (WES) was performed to isolate the causative gene of two unrelated neuropathy patients (9 and 13 years of age) with onset of the syndrome. Clinical assessments and biochemical analysis were performed.

**Results:**

A novel homozygous mutation (p.R41Q) in *MPV17* was found by WES in both patients. Both showed axonal sensorimotor polyneuropathy without liver and brain involvement, which is neurophysiologically similar to axonal Charcot-Marie-Tooth disease (CMT). A distal sural nerve biopsy showed an almost complete loss of the large and medium-sized myelinated fibers compatible with axonal neuropathy. An i*n vitro* assay using mouse motor neuronal cells demonstrated that the abrogation of *MPV17* significantly affected cell integrity. In addition, the expression of the mutant protein affected cell proliferation. These results imply that both the loss of normal function of *MPV17* and the gain of detrimental effects of the mutant protein might affect neuronal function.

**Conclusion:**

We report a novel homozygous mutation in *MPV17* from two unrelated patients harboring axonal sensorimotor polyneuropathy without hepatoencephalopathy. This report expands the clinical spectrum of diseases caused by mutations of *MPV17*, and we recommend *MPV17* gene screening for axonal peripheral neuropathies.

**Electronic supplementary material:**

The online version of this article (doi:10.1186/s12883-015-0430-1) contains supplementary material, which is available to authorized users.

## Background

Mutations in *MPV17* cause mitochondrial DNA depletion syndrome 6 (MTDPS6) (NNH; MIM #256810), also known as Navajo neurohepatopathy, an autosomal, recessive, multi-system disorder [1]. MTDPS6 is divided into three clinical phenotypes based on age at onset and the course of the disease: infantile form (onset before age of 6 months) with jaundice and failure to thrive before 2 years of age, childhood form (onset between age 1 and 5 years) resulting in early death from liver failure, and the classical form with moderate liver dysfunction and progressive neuropathy [2].

The function of *MPV17* protein remains uncertain. Further investigation is needed to determine whether the disease is primarily a neurologic disorder or a consequence of a metabolic disorder. *MPV17* encodes a mitochondrial, inner membrane protein, and it has been implicated in the metabolism of oxidative phosphorylation (OXPHOS), glycogen storage, mitochondrial morphology, and the integrity of mitochondrial DNA (mtDNA) under stress conditions [3]. An *MPV17-*deficient mouse model exhibited severe mtDNA depletion in skeletal muscle and the liver, but not in the brain or kidneys. Other phenotypes of NNH, such as liver cirrhosis or failure, are the most common, but have not occurred. However, the mice exhibited sensorineural deafness due to severe degeneration of the inner ear, and with associated apoptosis of the outer hair cells [4–6]. These results imply the significance of *MPV17* in cellular integrity. In addition, in the absence of *MPV17,* yeast cells became more vulnerable to metabolic or oxidative stresses [7].

Although the incidence of the neurohepatopathy by *MPV17* mutations in the western Navajo Reservation is relatively frequent (1 in 1,600 live birth), a few non-Navajo patients have been investigated [8, 9]. The presentation of NNH revealed multi-systemic disorder, and the patients with classical form showed axonal sensorimotor neuropathies with other clinical manifestations, such as liver disease, diabetic mellitus, or brain abnormalities.

Here, we report on two unrelated patients with *MPV17* homozygous mutation. In contrast to previously reported NNH patients [2, 8–11], the patients presented here exhibited only axonal sensorimotor polyneuropathy without liver and brain symptoms. This is the first report of *MPV17* dysfunction in Korean patients, and we analyzed the detrimental effect of mutant *MPV17* on a motor neuronal cell line.

## Methods

### Subjects

**Fig. 1:**
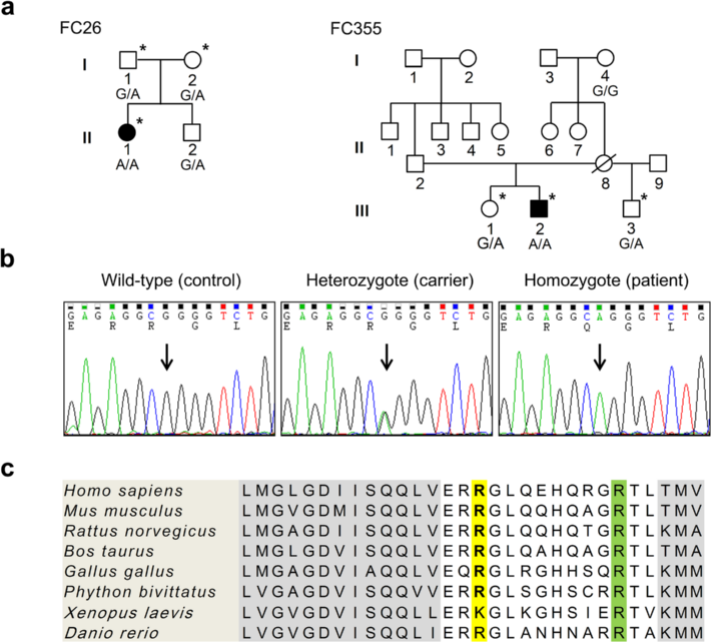
Pedigree, sequencing chromatograms, and conservation analysis. **a** Pedigrees of FC26 (*left*) and FC355 families (*right*). Genotypes of *MPV17* c.122G> A mutation were indicated bottom of each examined individuals. The open symbols represent unaffected individuals and filled symbols represent affected individuals. Asterisks indicate samples whose DNA were used for WES. **b** Confirmation of the mutation by capillary sequencing method. *Vertical arrows* indicate the mutation site. **c** Conservation analysis of mutation site in *MPV17*. The mutation site (R41, *yellow*) and adjacent amino acid sequences are well conserved across species. R50 and transmembrane domains are indicated in green and gray colors, respectively (*H. sapiens*: NP_002428.1, *M. musculus*: NP_032648.1, *R. norvegicus*: NP_001091710.1, *B. taurus*: NP_001039394.1, *G. gallus:* XP_004935875.1, *P. bivittatus*: XP_007420911.1, *X. laevis*: AAH82223.1, and *D. rerio*: NP_957459.2)

This study enrolled two autosomal recessive Korean families with axonal sensorimotor polyneuropathy (FC26 and FC355, Fig. 1a). After careful clinical and electrophysiological examinations, 300 healthy controls were recruited from the neurological department. All participants provided written, informed consents according to the protocol approved by the Institutional Review Board for Ewha Womans University, Mokdong Hospital (ECT 11-58-37). In addition, the patients provided written, informed consent for the publication of individual clinical details, and for the publication of family trees.

### Clinical assessments

Two independent neurologists evaluated each patient, and collected clinical information including assessments of motor and sensory impairments, deep tendon reflexes, and muscle atrophy. Muscle strength of flexor and extensor muscles were assessed manually using the standard medical research council (MRC) scale. In order to detect any physical disability we used a nine-point functional disability scale (FDS) [12], which was based on the following criteria: 0: normal; 1: normal but with cramps and fatigability; 2: an inability to run; 3: walking difficulty but still possible unaided; 4: walking with a cane; 5: walking with crutches; 6: walking with a walker; 7: wheelchair bound; and 8: bedridden. Sensory impairments were assessed for the level and severity of pain, temperature, vibration and position. Age at onset was determined by asking patients for their ages, when symptoms first appeared.

### Electrophysiological study

Motor and sensory conduction velocities of median, ulnar, peroneal, tibial, and sural nerves were determined. Motor conduction velocities (MCVs) of the median and ulnar nerves were determined by stimulating at the elbow and wrist, while recording compound muscle action potentials (CMAPs) over the abductor pollicis brevis and adductor digiti quinti, respectively. In the same way, the MCVs of peroneal and tibial nerves were determined by stimulating at the knee and ankle, while recording CMAPs over the extensor digitorum brevis and adductor hallucis, respectively. Sensory conduction velocities (SCVs) were obtained over a finger-wrist segment from the median and ulnar nerves by orthodromic scoring, and were also recorded for sural nerves. Sensory nerve action potential (SNAP) amplitudes were measured from positive peaks to negative peaks.

### Sural nerve biopsy

Distal sural nerve was biopsied from patient 1 (Fig. 1a, II-1) at 34 years, and pathological examination included light and electron microscopic analyses. Formalin-fixed sections were stained with hematoxylin and eosin (H&E), modified Masson’s trichrome, and Luxol fast blue. For electron microscopic study, the specimen was fixed in 2 % glutaraldehyde in 25 mM cacodylate buffer. Semithin sections were stained with toluidine blue and ultra-thin cut samples were contrasted with uranyl acetate and lead citrate.

### Vastus lateralis muscle biopsy

Cross-sections of the biopsy of the vastus lateralis muscle from patient two (Fig. 1b, III-2) at 22 years were stained with H&E, modified Gomori-trichrome, NADH-tetrazolium reductase (NADH-TR), succinate dehydrogenase (SDH), periodic acid Schiff (PAS), Oil-red-O and adaenosine triphosphatase reaction, and immunostained for myosin heavy chain (Vision Biosystems, Newcastle, UK). Samples were also examined using electron microscopy.

### MR images of brain, hip, thigh and lower leg

Brain, hip, thigh and lower leg of both patients were evaluated using a 1.5-T system (Siemens Vision; Siemens, Erlangen, Germany). Whole brains were scanned using a slice thickness of 7 mm and 2-mm interslice gap, to produce 16 axial images. The imaging protocol consisted of T2-weighted spin echo (SE) (TR/TE = 4,700/120 ms), T1-weighted SE (TR/TE = 550/12 ms), and fluid-attenuated inversion recovery (FLAIR) (TR/TE = 9,000/119 ms, inversion time 2,609 ms) images. Images of the hip, thigh and lower leg were obtained in axial [field of view (FOV) 24–32 cm, slice thickness 6 mm, and slice gap 0.5–1.0 mm] and coronal planes (FOV 38–40 cm, slice thickness 4–5 mm, slice gap 0.5–1.0 mm). xThe following protocol was used: T1-weighted SE (TR/TE 570–650/14–20, 512 matrices), T2-weighted SE (TR/TE 2800–4000/96–99, 512 matrices), and fat-suppressed T2-weighted SE (TR/TE 3090–4900/85–99, 512 matrices).

### Exome sequencing and filtering

Whole exome sequencing (WES) was performed for six samples (three from each family), according to a previous study [13]. Briefly, WES was performed using the Human SeqCap EZ Human Exome Library v3.0 (Roche/NimbleGen, Madison, WI, USA), and the HiSeq 2000 Genome Analyzer (Illumina, San Diego, CA, USA). The UCSC assembly hg19 was used as the reference sequence and variant calling was achieved in cases with >20 single nucleotide polymorphisms (SNP). We collected functionally significant variants (missense, nonsense, exonic indel and splicing site variants) from about 70 peripheral neuropathy genes and 15 mitochondrial DNA depletion syndrome (MTDPS)-related genes, and then variants agreeing with autosomal recessive inheritance were selected. Causative variants were confirmed by the Sanger’s sequencing method, and conservation analysis of mutation sites was performed using the MEGA5 program, ver 5.05 (http://www.megasoftware.net/). *In silico* analyses were performed using the prediction algorithms SIFT (http://sift.jcvi.org) and MUpro (http://www.ics.uci.edu/~baldig/mutation).

### Construction of wild-type and mutant *MPV17*

To obtain the *MPV17* transcript, cDNA was synthesized using Superscript reverse transcriptase (Invitrogen, Carlsbad, CA, USA) from total mRNA of HEK293. Then polymerase chain reaction (PCR) was performed using the cDNA as a template. The amplified PCR product was cloned into the expression vector, pCMV-myc (Clontech, Mountain View, CA, USA). Mutant *MPV17* transcript were generated by QuikChange Site-Directed Mutagenesis Kit (Stratagene, La Jolla, CA, USA). All primers’ sequences are listed in Additional file 1: Table S1.

### Transfection and knockdown of *MPV17*

NSC34 cells were cultured in a 10 % FBS, 1 % PS and high glucose Dulbecco’s modified eagle medium (DMEM; Biowest, Nuaille, France). To express *MPV17* transcript in the motor neuron, NSC34 cells were transfected with *MPV17* DNA-containing vectors using Lipofectamine 2000 reagent (Invitrogen), according to the manufacturer’s recommendation. Knockdown of *MPV17* was performed using *MPV17*-specific siRNA and Lipofectamine 2000 reagent (Invitrogen) (Additional file 1: Table S1). Cells were harvested after overexpression and knockdown of *MPV17* at 24 and 72 h.

### Measurement of proliferation and cell viability

After 3 days of knockdown, NSC34 cells were transferred to 24-well plates. Then, the proliferation of the cells was determined by direct counting under a microscope at 24 h intervals. For the overexpression model, NSC34 cells, cultured on 24-well plates, were transfected with wild-type or mutant *MPV17*. Cells were counted at 24 h intervals. Sensitivity to H_2_O_2_ was measured by a 3-(4,5-dimethylthiazol-2-yl)-2,5-diphenyltetrazolium bromide (MTT) assay. Briefly, cells treated with H_2_O_2_ were incubated with 10 mM MTT solution for 2 h, then the cells were lysed with dimethyl sulfoxide. Relative numbers of viable cells were determined using absorbance at 560 nm.

### Western blotting

Protein synthesis in NSC34 cells was determined using standard Western blotting with anti-myc Ab (Abcam, Cambridge, UK), anti-actin Ab, anti-mouse secondary Ab, and anti-rabbit secondary Ab (Sigma, St. Louis, MO, USA). An OXPHOS detection cocktail (Abcam) was used based on standard Western blotting. ECL plus Western blotting substrate (Thermo Scientific, Rockford, IL, USA) were used for detection of proteins.

## Results

### Identification of a novel homozygous mutation in *MPV17* gene

The mean sequencing yields of six WES data was approximately 11.18 Gb/sample with mappable reads of 96 %. Approximately, 89,235 variants (SNPs and indels) were observed from each sample. Of these, 20,775 were located in a coding region (Additional file 1: Table S2).

After filtering the WES data for 3 members in each family (Fig. 1), a novel homozygous mutation c.122G> A (p.R41Q) in *MPV17* was identified in both families (Fig. 1b). A mutant allele was putatively inherited from each parent in both families. The mutation was not observed in the 300 controls and in-house exome data (*n* = 302). In addition, it was not registered in the dbSNP142 (http://www.ncbi.nlm.nih.gov) or the 1000 Genomes Project Database (http://www.1000genomes.org/). However, this mutation was reported in the Exome Sequencing Project database (http://evs.gs.washington.edu/EVS/) and ExAc browser (http://exac.broadinstitute.org/) with very low allele frequency (0.00008 and 0.00002471, respectively). The mutation site was well-conserved across vertebrate species (Fig. 1c). *In silico* analysis of the mutation was predicted to affect the protein stability by SIFT (0.02) or MUPro (−0.422) programs. Although more than 40 functionally significant variants were found in ~70 CMT- and ~15 MTDPS-related genes, they were not considered the genetic cause, except for the *MPV17* mutation, because they were found in the controls or noncosegregated with affected individuals (Additional file 1: Table S3 and S4).

### Clinical manifestations

#### Patient 1

**Fig. 2:**
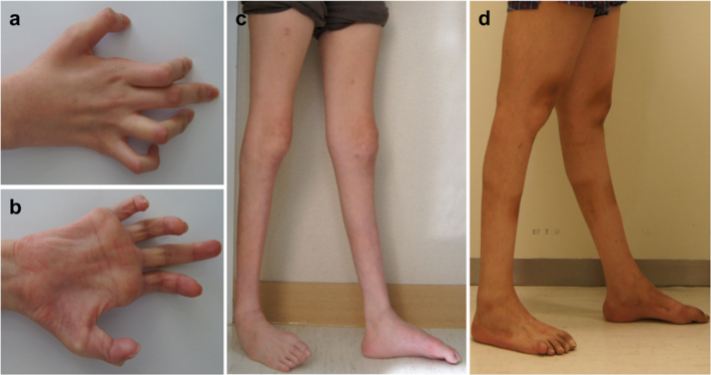
Hand and leg pictures of patients. **a**, **b** Hands of patient 1 (**a**) and 2 (**b**). Right hand showed severe, atrophied, intrinsic muscle and flexion deformities of interphalangeal joints. **c**, **d** Lower extremities of patient 1 (**c**) and 2 (**d**). Severe bilateral muscle atrophies and weakness with ankle joint deformities were observed in both patients

A 34-year-old woman (FC26; Fig. 1a, II-1) was the first child of healthy non-consanguineous Korean parents. The proband was born at full term and the perinatal histories were unremarkable. Early motor milestones were not delayed, and 1 year after her birth, she was able to walk. At 9 years of age, she first noticed muscle weakness of the distal lower limbs. She began to walk with short leg braces at 12 years of age. Neurologic examination at the age of 34 years revealed muscle weakness and atrophies of bilateral, distal muscles, predominantly in the lower limbs. Bilateral, severe atrophic changes of the intrinsic hand, foot and calf muscles, and flexion deformities of interphalangeal joints were noted (Fig. 2a-c). Ankle joint deformity and scoliosis was observed although she was able to walk with orthopedic assistance. Vibration and position senses were more severely disturbed than pain and touch senses. Knee and ankle jerks were absent. No pyramidal or cerebellar signs were detected. However, she did not present any other clinical presentations of NNH, such as growth retardation, gastrointestinal dysmotility, hepatomegaly, cognitive impairment, ophthalmoplegia, corneal scarring or hypoglycemic attacks. Elevated serum lactate levels were revealed (2.1 mmol/L, reference value: < 1.6 mmol/L), but the serum levels of liver enzyme, glucose and pyruvate were normal.

#### Patient 2

A 22-year-old man (FC355; Fig. 1b, III-2) was the second child of healthy non-consanguineous parents. Early motor milestones were normal, as in patient one. At age 13, he first felt gait and balance problems. He began to walk with short leg braces at 20 years of age. Neurologic examination at the age of 22 years revealed muscle weakness and atrophies of the bilateral distal muscles. Bilateral flat feet, and atrophic changes of intrinsic hand, foot and calf muscles were also noted (Fig. 2d). Like patient one, he complained that vibration and position senses were more severely disturbed than pain and touch senses, and he was able to walk with assistance. Deep tendon reflexes, in all extremities, were absent. He did not present with other phenotypes of NNH. Laboratory findings were normal except for a slightly elevated serum lactate (1.9 mmol/L).

### Sensory nerve involvement in the early stage of disease

An electrophysiological study showed similar results in both patients (Table 1). Sensory-nerve action potentials of the sural nerve were lost in the early stage of the disease. MCVs and CMAPs were decreased in median and ulnar nerves and CMAPs of peroneal and tibial nerves were not elicited. SCVs and SNAPs were not elicited in either patient. Visual evoked potential (VEP) and brainstem auditory evoked potential (BAEP) were normal.

**Table 1:** Electrophysiological features of the patients with *MPV17* mutation

	Patient 1							Patient 2				Normal value
Age at exam (years)	12	13	17	33		34		21		22		
Side	Rt	Rt	Rt	Rt	Lt	Rt	Lt	Rt	Lt	Rt	Lt	
Median nerve												
TL (ms)	3.7	**4.3**	**4.6**	**9.2**	**7.0**	**9.2**	**6.5**	3.6	3.7	3.2	3.1	<3.9
CMAP (mV)	10.0	7.7	**2.3**	**2.1**	**1.0**	**2.5**	**1.1**	15.1	13.3	13.9	10.9	>6.0
MNCV (m/s)	**39.6**	**41.3**	**57.1**	**39.0**	**38.6**	**41.1**	**41.5**	59.5	57.1	61.3	54.4	>50.5
Ulnar nerve												
TL (ms)	**3.3**	**3.4**	**3.2**	**9.9**	**6.4**	**9.2**	**6.0**	2.3	2.3	2.5	2.3	<3.0
CMAP (mV)	11.0	9.5	**6.3**	**0.8**	**3.8**	**0.7**	**3.0**	12.7	10.1	11.1	9.3	>8.0
MNCV (m/s)	51.7	**43.5**	**39.0**	**43.4**	**39.0**	**43.5**	**40.4**	56.1	62.6	56.9	60.5	>51.1
Peroneal nerve												
TL (ms)	**A**	**A**	**A**	**A**	**A**	**A**	**A**	**A**	**A**	**A**	**A**	<5.3
CMAP (mV)	**A**	**A**	**A**	**A**	**A**	**A**	**A**	**A**	**A**	**A**	**A**	>1.6
MNCV (m/s)	**A**	**A**	**A**	**A**	**A**	**A**	**A**	**A**	**A**	**A**	**A**	>41.2
Tibial nerve												
TL (ms)	**A**	**A**	**A**	**A**	**A**	**A**	**A**	**A**	**A**	**A**	**A**	<5.4
CMAP (mV)	**A**	**A**	**A**	**A**	**A**	**A**	**A**	**A**	**A**	**A**	**A**	>6.0
MNCV (m/s)	**A**	**A**	**A**	**A**	**A**	**A**	**A**	**A**	**A**	**A**	**A**	>41.1
Median sensory nerve												
SNAP (μV)	**A**	**A**	**A**	**A**	**A**	**A**	**A**	**A**	**A**	**A**	**A**	>8.8
SNCV (m/s)	**A**	**A**	**A**	**A**	**A**	**A**	**A**	**A**	**A**	**A**	**A**	>39.3
Ulnar sensory nerve												
SNAP (μV)	**A**	**A**	**A**	**A**	**A**	**A**	**A**	**A**	**A**	**A**	**A**	>7.9
SNCV (m/s)	**A**	**A**	**A**	**A**	**A**	**A**	**A**	**A**	**A**	**A**	**A**	>37.5
Sural nerve												
SNAP (μV)	**A**	**A**	**A**	**A**	**A**	**A**	**A**	**A**	**A**	**A**	**A**	>6.0
SNCV (m/s)	**A**	**A**	**A**	**A**	**A**	**A**	**A**	**A**	**A**	**A**	**A**	>32.1
H-reflex (ms)	**A**	**A**	**A**	**A**	**A**	**A**	**A**	**A**	**A**	**A**	**A**	<30.2

*Abbreviations*: *A* absent potentials, *TL* terminal latency, *CMAP* compound muscle action potential, *MNCV* motor nerve conduction velocity, *SNAP* sensory nerve action potential, *SNCV*, sensory nerve conduction velocity, *ND* not done. Bold character indicates abnormal values

### Fatty replacement in the soleus muscle

**Fig. 3:**
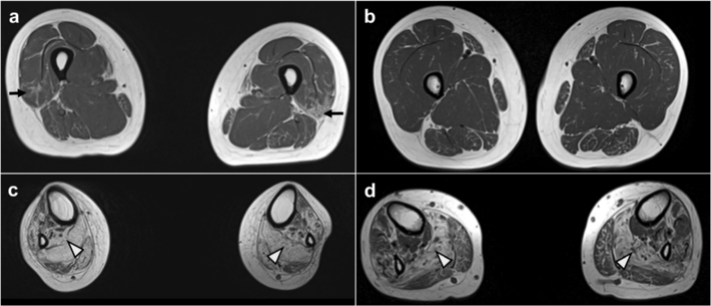
Lower extremity MRI of patients. **a, b** T1-weighted axial MRIs of the thigh and (**c, d**) lower leg of patient 1 (**a** and **c**) and 2 (**b** and **d**). At the thigh level, muscle atrophies and hyperintense signal changes were shown in the vastus lateralis muscle (*arrow*). However, the lower leg MRIs revealed diffuse fatty hyperintense signal changes in the soleus muscles (*arrowhead*), but the tibialis posterior and gastrocnemius muscles were relatively spared

MR imagery at the thigh level of patient one showed that the vastus lateralis muscle was mildly affected compared to other muscles (Fig. 3a, arrow), while nearly all muscles were normal in patient two (Fig. 3b). Lower calf muscle MRIs showed predominant and severe muscle atrophies and fatty replacements in the soleus muscles of both patients; however, the tibialis posterior and lateral gastrocnemius muscles were relatively sparing (Fig. 3c and d). It is noteworthy that both patients showed similar MRI patterns: T1-weighted images showed marked fatty infiltration in the lower calf muscles compared to the thigh and the hip, which was consistent with the length-dependent axonal degeneration. Brain MRI did not reveal any abnormality in either patient.

### Marked loss of large and medium sized myelinated fibers

**Fig. 4:**
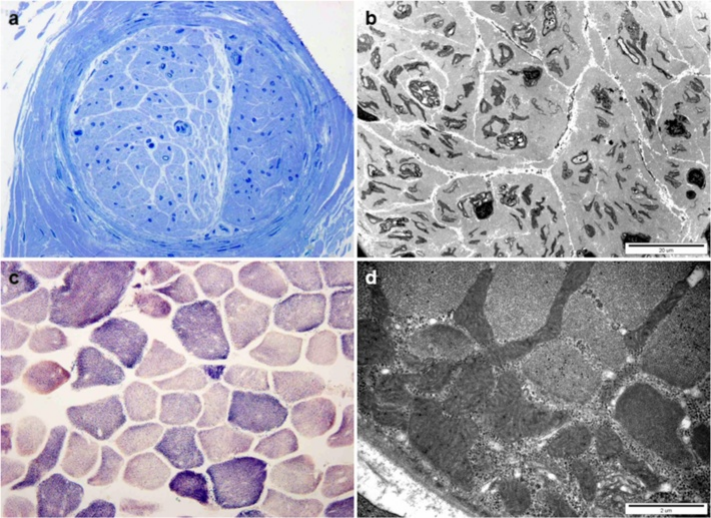
Histopathological characterization of sural nerve and vastus lateralis muscle. Sural nerve (**a** and **b**) from patient 1, and vastus lateralis muscle (**c** and **d**) biopsies from patient two were performed at 34 and 22 years, respectively. **a** Semi-thin transverse section. Toluidine blue stain shows the absence of large and medium myelinated fibers with rarely-noted, small myelinated fibers (x400). **b** Electron microscopic examination. It showed unmyelinated axons with atrophy and vesicular changes. **c** SDH reaction of skeletal muscle. Scattered myofibers showed increased positive reaction (x200). **d** Myofibers with focal subsarcolemmal accumulation of enlarged mitochondria

Semithin transverse sections from the left distal sural nerve biopsy, from patient 1, showed several remaining small myelinated fibers (MFs) with complete loss (10/mm^2^) of large and medium-sized MFs (normal distal sural nerve in 32-year-old female: 9,200/mm^2^) (Fig. 4a). Electron microscopic examination showed atrophy and occasional vesicular changes in the unmyelinated axons. There was no evidence of demyelination, remyelination or onion bulb formation (Fig. 4b).

### Chronic myopathy with few ragged red fibers

NADH-TR (data not shown) and SDH (Fig. 4c) stain in the left vastus lateralis muscle biopsy of patient two, showed increased irregular positive reaction, and modified Gomori trichrome stain (data not shown) showed a few ragged, red fibers. Electron microscopic examination showed myofibers with focal subsarcolemmal accumulation of enlarged mitochondria and abnormal membranous structure (Fig. 4d).

### Mutant protein inhibits cell proliferation and viability

**Fig. 5:**
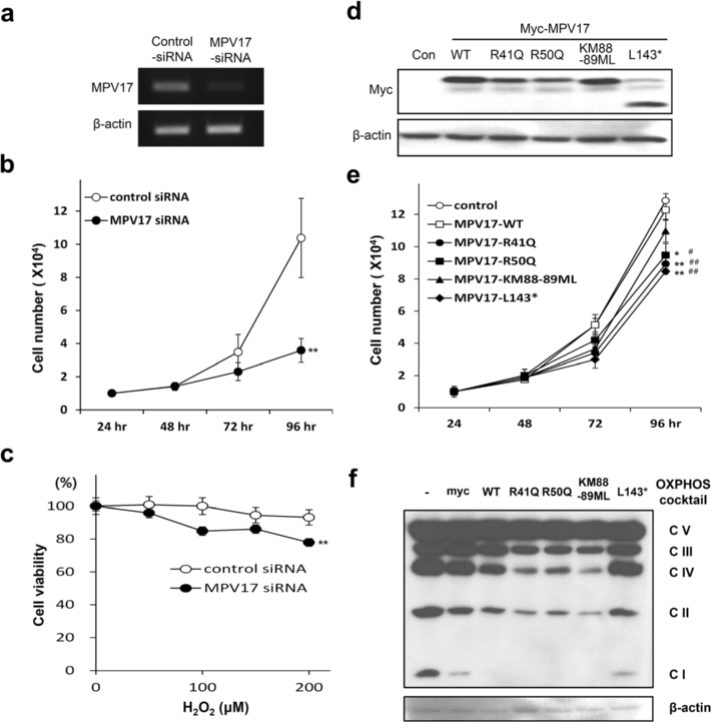
Effect of knockdown of *MPV17* and overexpression of mutant proteins on cell proliferation. **a** Confirmation of *MPV17 k*nockdown in NSC34 by RT-PCR. **b** Inhibitory effect of the proliferation of NSC34 by the abrogation of *MPV17* using specific siRNA. **c** Affection of *MPV17* mutations on cell viability against reactive oxygen species (ROS). Knockdown of *MPV17* using specific siRNA renders NSC34, a mouse motor neuronal cell line, more sensitive to H_2_O_2_ treatment (24 h). **d** Western blot analysis to determine the expression of mutant proteins. **e** Overexpression of mutant proteins affected cell proliferation of NSC34. **f** Changes in the mitochondrial OXPHOS system. Western blotting using OXPHOS detection cocktail antibody was performed after overexpression of wild-type and mutant *MPV17* proteins. For cell proliferation and survival assay, 4–6 wells per each sample were counted. Data are presented as mean ± SEM. Statistical analysis were performed using Student’s *t*-test. * and ^#^, *p* < 0.05; **and ^##^, *p* < 0.01. Statistical significance was determined either with control myc (* and **) or wild-type MPV17 (^#^ and ^##^) in (**e**)

To investigate the role of *MPV17* in motor neurons, we measured the effect on cell proliferation after abrogation of *MPV17*. Transfection of *MPV17*-specific siRNA for 72 h efficiently reduced the mRNA level in NSC34, a mouse motor neuronal cell line (Fig. 5a). In this setting, knockdown of *MPV17* significantly reduced the cell proliferation (Fig. 5b) and viability against ROS in mouse motor neuronal cells (Fig. 5c). Abrogation of MPV17 also affected mitochondrial integrity, which was observed by amounts of OXPHOS (Additional file 2: Figure S1a). Next, in order to investigate the effect of *MPV17* mutations, we cloned wild-type *MPV17* gene and generated mutations including p.R41Q. Expression of the proteins in NSC34 was confirmed by Western blotting (Fig. 5d). Expression of p.R41Q mutant protein significantly inhibited cell proliferation when compared to controls (Fig. 5e). In addition, expression of p.R50Q and p.L143* proteins significantly reduced cell proliferation, whereas p.KM88-89ML mutant exhibited a mild effect. Overexpression of the mutants mildly affected mitochondrial OXPHOS system in the presence of endogenous *MPV17* protein (Fig. 5f).

## Discussion

Clinical features of the present patients are considerably different from those of previously described NNH patients with *MPV17* mutations. The disease presentation has frequently been described as axonal sensorimotor neuropathies; however, the neuropathies were always associated with hepatopathy, or brain involvements, in the classical form [14-15]. But, the present two patients showed only axonal sensorimotor polyneuropathy, and they did not exhibit any liver or brain abnormalities, at the ages of 34 and 22 years. However, these patients are young and other organ systems may eventually become involved, so follow-up monitoring is needed. We also observed severe impairment of the hands and lower legs with severe muscle atrophy and contractures due to peripheral neuropathy. In addition, lower extremity MRI revealed severe distal fatty infiltration, which is consistent with length-dependent axonal degeneration. Moreover, the lower leg MRIs exhibit selective fatty infiltration with a preference for soleus muscles, similar to CMT type 2A (CMT2A) caused by *MFN2* mutations [16].

Currently, there is limited information on the function of *MPV17*. The *MPV17*^−/−^ mouse model exhibited peripheral neuropathy, with sensorineural deafness and kidney failure [6, 17], which is in good agreement with the human phenotype. In addition, the loss of *MPV17* resulted in apoptosis in outer hair cells, which implies the significance of *MPV17* in cellular viability [4–6]. To address the function of *MPV17* in the peripheral nervous system (PNS), we first confirmed the expression of *MPV17* in human PNS using transcriptome data from human sural nerve (data not shown). We observed that *MPV17* abrogation significantly affects the mitochondrial OXPHOS system, susceptibility to ROS, and cell proliferation. These data suggest that sustained expression of *MPV17* is critical to PNS.

To address the effect of the p.R41Q mutation, we compared the effect of the mutant protein on cell viability with several previously reported mutants. Structurally, p.R41Q mutation is similar to p.R50Q in that both amino acids are located in the matrix region between the first two transmembrane regions [1, 2, 18]. However, the clinical phenotype is quite different [3, 14]. In addition, the phenotype of a compound heterozygote mutation, p.KM88-89ML and p.L143*, is closest to that of the present mutation, although the patient exhibited a fatty liver and hearing loss [15]. Analysis of the cell proliferation revealed that overexpression of the present mutation exhibited a negative effect, similar to p.R50Q and p.L143* mutation. In addition, several mutant proteins mildly affected the mitochondrial OXPHOS system. The mitochondrial oxidative phosphorylation system was affected by the expression of mutant proteins or partly by wild-type MPV17. This result is consistent with a previous report that liver samples, from *MPV17* mutation (P64R) harboring patients, revealed low levels of complex I, III and IV subunits. Although we could not determine the effect on the integrity of mitochondrial DNA (mtDNA) using patients’ samples, we tried with overexpression model. There was no mtDNA deletion, however, we observed that overexpression of wild-type or mutant MPV17 induced mtDNA depletion (Additional file 2: Figure S1b). These data suggest that overexpression of MPV17 might cause detrimental effect.

Recently, Uusimaa et al. reported two cases of a new mutation at the same amino acid (p.R41W) in *MPV17* [9]. The phenotype of the patient was milder than the aforementioned patients who exhibited progressive, neurological deterioration. They did not exhibited any defect in muscle, liver cirrhosis nor focal fibrosis. Experimentally, we observed that overexpression of R41W mutant in NSC34 cell also affects cellular proliferation (Additional file 2: Figure S1c). Thus these data implicate that mutation at Arg^41^ predominantly cause peripheral neuropathy.

## Conclusion

We suggest that a novel homozygous p.R41Q mutation in *MPV17* causes axonal sensorimotor polyneuropathy without hepatoencephalopathy. Our observations will expand the clinical spectrum of *MPV17*-causing disease and suggest that *MPV17* should be considered in screening tests for patients presenting only with axonal sensorimotor neuropathy.

## Additional files

Additional file 1:
**Table S1.** List of primers and siRNAs. **Table S2.** Summary of exome sequencing data. **Table S3.** List of CMT- and MTDPS- related genes. **Table S4.** Polymorphic nonsynonymous variants in peripheral neuropathy- and mitochondrial DNA depletion syndrome- related genes from the exome date. (DOCX 43 kb) Additional file 2: Figure S1. (a) Changes in the mitochondrial OXPHOS system. Western blotting using OXPHOS detection cocktail antibody was performed after knockdown of *MPV17*. Data are presented as mean ± SEM. *, *p* < 0.05; **, *p* < 0.01. (b) Mitochondrial DNA (mtDNA) depletion assay. HEK293 cells were treated with control (pCMV-myc), wild-type or mutant MPV17 transcripts for 72 days, then total DNA was purified and mtDNA depletion was analyzed by comparing the ratio of genomic DNA (beta actin) and mtDNA (ND1 and COX1) using realtime PCR. (c) Overexpression of mutant proteins affected cell proliferation of NSC34. Both MPV17 mutant proteins, R41Q and R41W, affected cell proliferation. (TIFF 2825 kb)

## Abbreviation

MTDPS6: Mitochondrial DNA depletion syndrome 6
NNH: Navajo neurohepatopathy
WES: Whole exome sequencing
OXPHOS: Oxidative phosphorylation
mtDNA: Mitochondrial DNA
CMT: Charcot-Marie-Tooth disease
CMT2A: Charcot-Marie-Tooth disease type 2A
MRC: Medical research council
MCVs: Motor conduction velocities
CMAPs: Compound muscle action potentials
SCVs: Sensory conduction velocities
SNAP: Sensory nerve action potential
NADH-RT: NADH-tetrazolium reductase
SDH: Succinate dehydrogenase
PAS: Periodic acid Schiff
SNP: Single nucleotide polymorphism
MTT: 3-(4,5-dimethylthiazol-2-yl)-2,5-diphenyltetrazolium bromide
VEP: Visual evoked potential
BAEP: Brainstem auditory evoked potential
MRI: Magnetic resonance imaging
