# Supplementary material for: A novel homozygous MPV17 mutation in two families with axonal sensorimotor polyneuropathy
Source: BMC Neurol. 2015 Oct 5;15:179. doi: 10.1186/s12883-015-0430-1 (PMC4595119; doi:10.1186/s12883-015-0430-1)
Supplement: Additional file 1: — Table S1. List of primers and siRNAs. Table S2. Summary of exome sequencing data. Table S3. List of CMT- and MTDPS- related genes. Table S4. Polymorphic nonsynonymous variants in peripheral neuropathy- and mitochondrial DNA depletion syndrome- related genes from the exome date. (DOCX 43 kb) [file 12883_2015_430_MOESM1_ESM.docx › updated additional files/1855616014153597_add1.docx]

**Additional file**

**Table S1** List of primers and siRNAs.

| Experiment | Primer name | Sequence |
| --- | --- | --- |
| cDNA cloning | MPV17-F | 5’-GCTCAGGAAGCATGGCACTCT-3’ |
|  | MPV17-R | 5’-CAGGCTTAGAGCCGATGTG-3’ |
| Mutagenesis | MPV17-R41Q-F | 5’-CTGGTGGAGAGGCAGGGTCTGCAGGA-3’ |
|  | MPV17-R41Q-R | 5’-CTGCAGACCCTGCCTCTCCACCAGCTG-3’ |
|  | MPV17-R41W-F | 5’-CTGGTGGAGAGGTGGGGTCTGCAGGA-3’ |
|  | MPV17-R41W-R | 5’-CTGCAGACCCCACCTCTCCACCAGCTG-3’ |
|  | MPV17-R50Q-F | 5’-cagagaggccAgactctgaccatgg-3’ |
|  | MPV17-R50Q-R | 5’-GGTCAGAGTCTGGCCTCTCTGGTGT-3’ |
|  | MPV17-KM88-89ML-F | 5’-tgcactgaagaTgTtgttgttggatcagg-3’ |
|  | MPV17-KM88-89ML-R | 5’-ATCCAACAACAACATCTTCAGTGCATCC-3’ |
|  | MPV17-L143*-F | 5’-gctgtgcagtGagccaacttctacc-3’ |
|  | MPV17-L143*-R | 5’-TAGAAGTTGGCTCACTGCACAGCAGGCC-3’ |
| Detection of mRNA level | mβActin-F | 5’-GTGACGTTGACATCCGTAAAGA-3’ |
|  | mβActin-R | 5’- TGCTAGACGTTGGTAGCTCCT-3’ |
|  | mMPV17-F | 5’-TGGCGAGCATACCAGAGAG-3’ |
|  | mMPV17-R | 5’-GGTCTAAAACTTTGTACCAGCCT-3’ |
|  | mEphx1-F | 5’-GGAGACCTTACCACTTGAAGATG-3’ |
|  | mEphx1-R | 5’-GCCCGGAACCTATCTATCCTCT-3’ |
|  | mAcot13-F | 5’-AGCAGCATGACCCAGAACCTA-3’ |
|  | mAcot13-R | 5’-GGAGCGTGCCCAGTTTATTAGTA-3’ |
|  | mGsta3-F | 5’-AAGAATGGAGCCTATCCGGTG-3’ |
|  | mGsta3-R | 5’-CCATCACTTCGTAACCTTGCC-3’ |
|  | mOpa1-F | 5’-CGACTTTGCCGAGGATAGCTT-3’ |
|  | mOpa1-R | 5’-CGTTGTGAACACACTGCTCTTG-3’ |
| Knockdown in NSC34 | Control-siRNA | 5’-CCUACGCCACCAAUUUCGUdTdT-3’ |
|  | mMPV17-1 siRNA | 5’-GAAGGCACAUCAGUUCUAAdTdT-3’ |
|  | mMPV17-2 siRNA | 5’-GCACGGAUUAUCUGCUCUAdTdT-3’ |

**Table S2** Summary of exome sequencing data.

| Samples |  | FC26 |  |  |  | FC355 |  |
| --- | --- | --- | --- | --- | --- | --- | --- |
|  | I-1 | I-2 | II-1 |  | III-1 | III-2 | III-3 |
| Total yields (Gbp) | 11.78 | 13.46 | 13.51 |  | 12.38 | 7.54 | 8.42 |
| Mappable reads (%) | 98.2 | 99.0 | 99.2 |  | 99.3 | 81.0 | 99.3 |
| Coverage of the target region (≥ 1X, %) | 95.1 | 95.3 | 95.3 |  | 96.0 | 97.6 | 95.2 |
| Coverage of the target region (≥ 10X, %) | 89.4 | 89.8 | 90.9 |  | 91.3 | 93.5 | 87.1 |
| Mean read depth of the target region (X) | 38.8 | 49.8 | 50.9 |  | 46.0 | 103.9 | 37.0 |
| Total number of SNPs | 85,309 | 86,977 | 87,599 |  | 87,386 | 56,687 | 82,681 |
| Number of coding SNPs | 20,351 | 20,519 | 20,545 |  | 20,737 | 19,574 | 20,329 |
| Total number of indels | 7,831 | 7,933 | 7,988 |  | 7,925 | 9,718 | 7,377 |
| Number of coding indels | 404 | 418 | 408 |  | 446 | 532 | 386 |

**Table S3.** List of CMT- and MTDPS- related genes

| Gene | MIM No | Type (phenotype) | Inheritance | Map |
| --- | --- | --- | --- | --- |
| CMT-related genes | |  |  |  |
| *PLEKHG5* | 611101 | LMND | AD, AR | 1p36.31 |
| *KIF1B* | 605995 | CMT2A1 | AD | 1p36.22 |
| *MFN2* | 608507 | CMT2A2, HMSN6 | AD | 1p36.22 |
| *YARS* | 603623 | DI-CMTC | AD | 1p35.1 |
| *NGFB* | 162030 | HSAN5 | AR | 1p13.2 |
| *LMNA* | 150330 | CMT2B1 | AD, AR | 1q22 |
| *NTRK1* | 191315 | HSAN4 | AR | 1q23.1 |
| *MPZ* | 159440 | CMT1B, 2I, 2J, DSS | AD | 1q23.3 |
| *DCTN1* | 601143 | dHMN7 | AD | 2p13.1 |
| *REEP1* | 609139 | dHMN5, HSP | AD | 2p11.2 |
| *TFG* | 602498 | HMSN-P | AD | 3q12.2 |
| *RAB7* | 602298 | CMT2B | AD | 3q21.3 |
| *GNB4* | 610863 | DI-CMTF | AD | 3q26.33 |
| *CCT5* | 610150 | HSAN with spastic paraplegia | AR | 5p15.2 |
| *FAM134B* | 613114 | HSAN2 | AR | 5p15.1 |
| *HSPB3* | 604624 | dHMN2C | AD | 5q11.2 |
| *SH3TC2* | 608206 | CMT4C | AR | 5q32 |
| *FIG4* | 609390 | CMT4J | AR | 6q21 |
| *GARS* | 600287 | CMT2D, dHMN5 | AD | 7p14.3 |
| *HSPB1* | 602195 | CMT2F, dHMN2B | AD | 7q11.23 |
| *ARHGEF10* | 608136 | Slowed NCV | AD | 8p23.3 |
| *NEFL* | 162280 | CMT1F, CMT2E | AD | 8q21.2 |
| *GDAP1* | 606598 | CMT2K, CMT4A, RI-CMTA | AD, AR | 8q21.11 |
| *NDRG1* | 605262 | CMT4D (HMSNL) | AR | 8q24.22 |
| *SPTLC1* | 605712 | HSAN1A | AD | 9q21.31 |
| *IKBKAP* | 603722 | HSAN3 | AR | 9q31.3 |
| *LRSAM1* | 610933 | CMT2P | AD, AR | 9q33.3 |
| *SETX* | 608465 | dHMN, ALS4 | AD, AR | 9q34.13 |
| *EGR2* | 129010 | CMT1D, DSS, CHN | AD | 10q21.3 |
| *SBF2* | 607697 | CMT4B2 | AR | 11p15.4 |
| *BSCL2* | 606158 | dHMN, Silver syndrome | AD | 11q12.3 |
| *IGHMBP2* | 600502 | dHMN6 | AR | 11q13.3 |
| *MTMR2* | 603557 | CMT4B1 | AR | 11q21 |
| *WNK1* | 605232 | HSAN2A | AR | 12p13.33 |
| *FGD4* | 611104 | CMT4H | AR | 12p11.21 |
| *TRPV4* | 605427 | CMT2C | AD | 12q24.11 |
| *HSPB8* | 608014 | CMT2L, dHMN2A | AD | 12q24.23 |
| *ATL1* | 606439 | HSAN1D, spastic paraplegia | AD | 14q22.1 |
| *SPTLC2* | 605713 | HSAN1C | AD | 14q24.3 |
| *TDP1* | 607198 | SCAN1 (CMT2) | AR | 14q32.11 |
| *DYNC1H1* | 600112 | CMT2O | AD | 14q32.31 |
| *SLC12A6* | 604878 | ACCPN | AR | 15q14 |
| *LITAF* | 603795 | CMT1C | AD | 16p13.13 |
| *AARS* | 601065 | CMT2N | AD | 16q22.1 |
| *KARS* | 601421 | RI-CMTB | AD | 16q23.1 |
| *GAN* | 605379 | GAN1 | AR | 16q23.2 |
| *PMP22* | 601097 | CMT1A, DSS, HNPP | AD | 17p12 |
| *SEPT9* | 604061 | HNA | AD | 17q25.2-q25.3 |
| *CTDP1* | 604927 | CCFDN | AR | 18q23 |
| *DNMT1* | 126375 | HSAN1E | AD | 19p13.2 |
| *DNM2* | 602378 | CMT2M, DI-CMTB | AD | 19p13.2 |
| *PRX* | 605725 | CMT4F, DSS | AR | 19q13.2 |
| *DMPK* | 605377 | DI-CMT with DM | AD | 19q13.32 |
| *MED25* | 610197 | CMT2B2 | AR | 19q13.33 |
| *MYH14* | 608568 | PNMHH | AD | 19q13.33 |
| *SOX10* | 602229 | CMT1 with Waardenburg syndrome | AD | 22q13.1 |
| *PDK3* | 300906 | CMTX6 | XD | Xp22.11 |
| *GJB1* | 304040 | CMTX1 | XD | Xq13.1 |
| *PRPS1* | 311850 | CMTX5 | XR | Xq22.3 |
| MTDPS-related genes | | | | |
| *SUCLG1* | 611224 | MTDPS9 | AR | 2p11.2 |
| *DGUOK* | 601465 | MTDPS3 | AR | 2p13.1 |
| *MPV17* | 137960 | MTDPS6, | AR | 2p23.3 |
| *SLC25A4* | 103220 | CMH1, PEOA2, MTDPS12 | AD, AR | 4q35.1 |
| *FBXL4* | 605654 | MTDPS13 | AR | 6q16.1-q16.3 |
| *AGK* | 610345 | CATC5, Sengers syndrome (MTDPS10) | AR | 7q34 |
| *RRM2B* | 604712 | MTDPS8A, MTDP8B, PEOA5 | AD, AR | 8q22.3 |
| *SURF1* | 185620 | Leigh syndrome, MTDPS3 | AR | 9q34.2 |
| *C10orf2* | 606075 | MTDPS7, IOSCA, PEOA3 | AD, AR | 10q24.31 |
| *SUCLA2* | 603921 | MTDPS5 | AR | 13q14.2 |
| *POLG* | 174763 | MTDPS4A, MTDPS4B, PEOA1, PEOB | AD, AR | 15q26.1 |
| *GFER* | 600924 | Myopathy, congenital cataract, hearing loss | AR | 16p13.3 |
| *TK2* | 188250 | MTDPS2 | AR | 16q21 |
| *POLG2* | 604983 | PEOA4 | AD | 17q23.3 |
| *C20orf72* | 615076 | MTDPS11 | AR | 20p11.23 |
| *TYMP* | 131222 | MTDPS1 | AR | 22q13.33 |

ACCPN: Agenesis of the corpus callosum with peripheral neuropathy, AD: autosomal dominant, ALS: amyotrophic lateral sclerosis, AR: autosomal recessive, CATC: cataract autosomal recessive congenital, CCFDN: congenital cataracts with facial dysmorphism and neuropathy, CHN: congenital hypomyelinating neuropathy, CMH: cardiomyopathy familial hypertrophic, CMT: Charcot-Marie-Tooth disease, dHMN: distal hereditary motor neuropathy, DI: dominant intermediate, DM: myotonic dystrophy, DSS: Dejerine-Sottas syndrome, FSGS: focal segmental glomerulosclerosis, GAN: giant axonal neuropathy, HMSN: hereditary motor and sensory neuropathy, HNA: hereditary neuralgic amyotrophy, HNPP: hereditary neuropathy with liability to pressure palsies, HSAN: hereditary sensory and autonomic neuropathy, HSN: hereditary sensory neuropathy, IOSCA: infantile-onset spinocerebellar ataxia, LMND: low motor neuron disease, MTDPS: mitochondrial DNA depletion syndrome, NCV: nerve conduction velocity, PN: peripheral neuropathy, PEO: progressive external ophthalmoplegia with mitochondrial DNA deletions, PNMHH: peripheral neuropathy, myopathy, hoarseness, and hearing loss, RI: recessive intermediate, SCAN: spinocerebellar ataxia, autosomal recessive with axonal neuropathy, XD: X-linked dominant, XR: X-linked recessive.

**Table S4.** Polymorphic nonsynonymous variants in peripheral neuropathy- and mitochondrial DNA depletion syndrome- related genes from the exome date

| Gene | Reference sequence^a^ | Variant | | dbSNP142 | 1000G^c^ | EVS^d^ | Family | Description^e^ |
| --- | --- | --- | --- | --- | --- | --- | --- | --- |
|  |  | Nucleotide^b^ | Amino acid |  |  |  |  |  |
| *MPZ* | NM_000530.6 | c.352G>A | p.D118N | - | - | - | FC26 | NC |
| *PLEKHG5* | NM_020631.4 | c.2428G>A | p.G810S | rs76625876 | 0.04 | 0.05 | FC26 | Pol, NC |
|  |  | c.1318A>G | p.M440V | rs61740145 | 0.05 | 0.06 | FC26 | Pol, NC |
| *FIG4* | NM_014845.5 | c.1090A>T | p.M364L | rs2295837 | 0.10 | 0.03 | FC26, FC355 | Pol, NC |
|  |  | c.1961T>C | p.V654A | rs9885672 | 0.37 | 0.33 | FC26, FC355 | Pol, NC |
| *ARHGEF10* | NM_014629.2 | c.3960G>C | p.R1320S | rs117084443 | 0.01 | - | FC26 | Pol |
| *NEFL* | NM_006158.4 | c.1413delC | [p.P471fs](http://www.ncbi.nlm.nih.gov/nuccore/NT_167187.1?report=graph&db=nucleotide&v=12669160:12669260&content=5&m=12669211!&mn=rs11300136) | rs11300136 | 0.99 | 1.00 | FC26,FC355 | Pol, NC |
| *IKBKAP* | NM_003640.3 | c.3473C>T | p.P1158L | rs1538660 | 0.22 | 0.21 | FC26,FC355 | Pol, NC |
|  |  | c.3214T>A | p.C1072S | rs3204145 | 0.22 | 0.21 | FC26,FC355 | Pol, NC |
|  |  | c.2605C>T | p.P869S | - | - | - | FC26 | NC |
|  |  | c.2490A>G | p.I830M | rs2230794 | 0.08 | 0.04 | FC26,FC355 | Pol, NC |
|  |  | c.2446A>C | p.I816L | rs2230793 | 0.29 | 0.27 | FC26,FC355 | Pol, NC |
|  |  | c.2417A>G | p.Y806C | rs191464698 | 0.00 | - | FC26 | Pol, NC |
|  |  | c.2294G>A | p.G765E | rs2230792 | 0.28 | 0.26 | FC26,FC355 | Pol, NC |
| *LRSAM1* | NM_138361.5 | c.952A>G | p.N318D | rs1539567 | 0.74 | 0.74 | FC26,FC355 | Pol, NC |
| *SETX* | NM_015046.5 | c.7834A>G | p.S2612G | rs3739927 | 0.16 | 0.07 | FC26,FC355 | Pol, NC |
|  |  | c.7759A>G | p.I2587V | rs1056899 | 0.51 | 0.44 | FC26,FC355 | Pol, NC |
|  |  | c.5563A>G | p.T1855A | rs2296871 | 0.41 | 0.31 | FC26,FC355 | Pol, NC |
|  |  | c.4156A>G | p.I1386V | rs543573 | 0.59 | 0.69 | FC355 | Pol, NC |
|  |  | c.3576T>G | p.D1192 | rs1185193 | 0.66 | 0.79 | FC355 | Pol, NC |
|  |  | c.1979C>G | p.A660G | rs882709 | 0.21 | 0.11 | FC355 | Pol |
| *IGHMBP2* | NM_002180.2 | c.602T>C | p.L201S | rs560096 | 0.70 | 0.78 | FC26,FC355 | Pol, NC |
|  |  | c.2011A>G | p.T671A | rs622082 | 0.22 | 0.25 | FC26, | Pol, NC |
|  |  | c.2636C>A | p.T879K | rs17612126 | 0.23 | 0.21 | FC26,FC355 | Pol, NC |
| *MTMR2* | NM_016156.5 | c.8A>C | p.K3T | rs3824874 | 0.28 | 0.24 | FC355 | Pol |
| *WNK1* | NM_213655.4 | c.2175_2176insC | p.P725fs | rs35706572 | 0.19 | - | FC26 | Pol, NC |
|  |  | c.2220_2221insC | p.L740fs | rs397768556 | 0.55 | - | FC26 | Pol, NC |
|  | NM_018979.3 | c.1994C>T | p.T665I | rs2286007 | 0.07 | 0.06 | FC26 | Pol, NC |
|  |  | c.3166A>C | p.T1056P | rs956868 | 0.85 | 0.85 | FC26,FC355 | Pol, NC |
|  |  | c.4517G>C | p.C1506S | rs7955371 | 0.99 | 0.99 | FC26,FC355 | Pol, NC |
|  |  | c.5424G>T | p.M1808I | rs12828016 | 0.39 | 0.43 | FC26,FC355 | Pol, NC |
| *SUCLA2* | NM_003850.2 | c.595T>A | p.S199T | rs7320366 | 0.72 | 0.74 | FC26,FC355 | Pol, NC |
| *INF2* | NM_022489.3 | c.3404C>T | p.T1135M | rs3803311 | 0.01 | 0.00 | FC355 | Pol, NC |
| *POLG* | NM_002693.2 | c.2890>T | p.R964C | rs201477273 | 0.00 | - | FC26 | Pol, NC |
| *GFER* | NM_005262.2 | c.457C>T | p.L153L | rs1046502 | 0.23 | 0.21 | FC26,FC355 | Pol, NC |
| *GAN* | NM_022041.3 | c.1239C>G | p.I413M | - | - | - | FC355 | NC |
| *POLG2* | NM_007215.3 | c.605A>G | p.K202R | - | - | - | FC26 | NC |
| *SEPT9* | NM_006640.4 | c.1672A>G | p.M558V | rs2627223 | 0.92 | 0.89 | FC26,FC355 | Pol, NC |
| *CTDP1* | NM_004715.4 | c.1019C>T | p.T340M | rs2279103 | 0.11 | 0.13 | FC26, | Pol, NC |
| *DNMT1* | NM_001379.2 | c.931A>G | p.I311V | rs2228612 | 0.19 | 0.09 | FC355 | Pol, NC |
| *PRX* | NM_181882.2 | c.3394G>A | p.G1132R | rs268674 | 0.96 | 0.95 | FC26,FC355 | Pol, NC |
| *DMPK* | NM_004409.4 | c.1267C>G | p.L423V | rs527221 | 0.12 | 0.10 | FC26 | Pol, NC |
| *ATP7A* | NM_000052.5 | c.4048G>A | p.E1350K | rs4826245 | 1.00 | - | FC26,FC355 | Pol, NC |

^a^GenBank registration number of reference sequence.

^b^cDNA numbering was achieved with +1, corresponding to the A of the ATG initiation codon.

^c^Variant allele frequencies in the 1000 Genomes database, March, 2014 (http://www.1000genomes.org/)

^d^Variant allele frequencies in the Exome variant server database (http://evs.gs.washington.edu/EVS/)

^e^Pol: polymorphism; NC: noncosegregation.
