# Supplementary figures and images for: A novel homozygous MPV17 mutation in two families with axonal sensorimotor polyneuropathy
Source: BMC Neurol. 2015 Oct 5;15:179. doi: 10.1186/s12883-015-0430-1 (PMC4595119; doi:10.1186/s12883-015-0430-1)

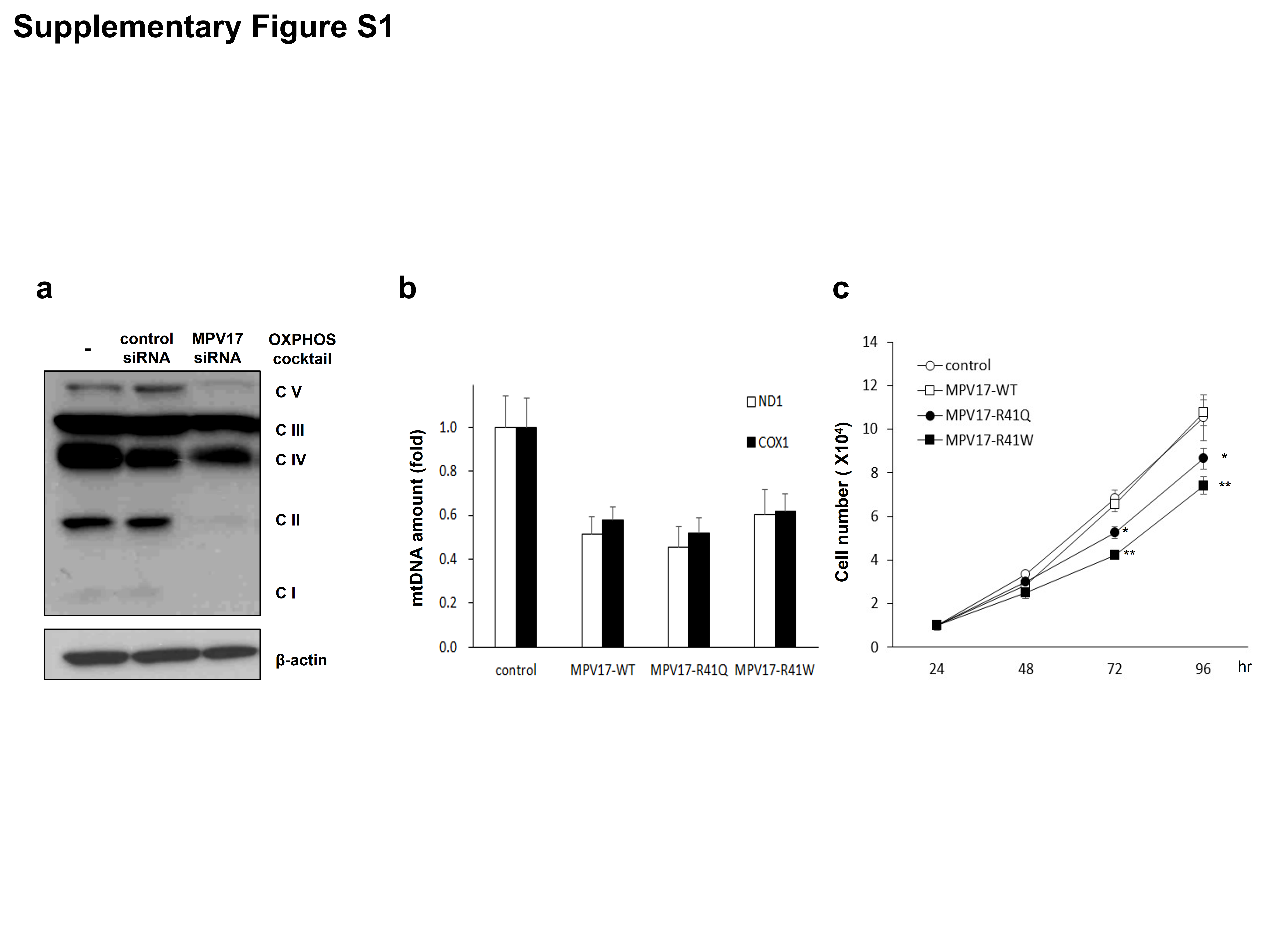

Supplement: Additional file 1: — Table S1. List of primers and siRNAs. Table S2. Summary of exome sequencing data. Table S3. List of CMT- and MTDPS- related genes. Table S4. Polymorphic nonsynonymous variants in peripheral neuropathy- and mitochondrial DNA depletion syndrome- related genes from the exome date. (DOCX 43 kb) [file 12883_2015_430_MOESM1_ESM.docx › updated additional files/1855616014153597_add2.tiff]
